# Altered functional hubs and connectivity in type 2 diabetes mellitus with and without mild cognitive impairment

**DOI:** 10.3389/fneur.2022.1062816

**Published:** 2022-12-12

**Authors:** Yang Huang, Dongsheng Zhang, Xin Zhang, Miao Cheng, Zhen Yang, Jie Gao, Min Tang, Kai Ai, Xiaoyan Lei, Xiaoling Zhang

**Affiliations:** ^1^Department of MRI, Shaanxi Provincial People's Hospital, Xi'an, China; ^2^Department of Clinical and Technical Support, Philips Healthcare, Xi'an, China

**Keywords:** type 2 diabetes mellitus, resting-state functional magnetic resonance imaging, degree centrality, functional connectivity, neuroimaging

## Abstract

Cognitive impairment in type 2 diabetes mellitus (T2DM) is associated with functional and structural abnormalities of brain networks, especially the damage to hub nodes in networks. This study explored the abnormal hub nodes of brain functional networks in patients with T2DM under different cognitive states. Sixty-five patients with T2DM and 34 healthy controls (HCs) underwent neuropsychological assessment. Then, degree centrality (DC) analysis and seed-based functional connectivity (FC) analysis were performed to identify the abnormal hub nodes and the FC patterns of these hubs in T2DM patients with mild cognitive impairment (MCI) (DMCI group, *N* = 31) and without MCI (DMCN group, *N* = 34). Correlation analyzes examined the relationship between abnormal DC and FC and clinical/cognitive variables. Compared with HCs, both T2DM groups showed decreased DC values in the visual cortex, and the T2DM patients with MCI (DMCI) showed more extensive alterations in the right parahippocampal gyrus (PHG), bilateral posterior cingulate cortex (PCC), and left superior frontal gyrus (SFG) regions than T2DM patients with normal cognitive function. Seed-based FC analysis of PHG and PCC nodes showed that functional disconnection mainly occurred in visual and memory connectivity in patients with DMCI. Multiple abnormal DC values correlated with neuropsychological tests in patients with T2DM. In conclusion, this study found that the DMCI group displayed more extensive alterations in hub nodes and FC in vision and memory-related brain regions, suggesting that visual-related regions dysfunctions and disconnection may be involved in the neuropathology of visuospatial function impairment in patients with DMCI.

## Introduction

Type 2 diabetes mellitus (T2DM) is a common systemic metabolic disease characterized by chronic hyperglycemia and insulin resistance (1). Long-term hyperglycemia can lead to various cognitive dysfunctions involving attention, memory, and visual space in patients with T2DM (2, 3). In addition, between 10.8% and 17.5% of patients with T2DM eventually develop mild cognitive impairment (MCI) and dementia (4). However, the neural mechanism of cognitive impairment in T2DM remains unclear. Abnormal changes in brain structure and function are the neural basis of cognitive impairment, especially damage to brain hub nodes and networks (5). Studies (6, 7) have found that cognitive dysfunction associated with different stages of diabetes have different features, and cognitive impairment caused by hyperglycemia may involve a complex process (8). Therefore, it is necessary to explore the patterns of brain function change in patients with T2DM under different cognitive states and comprehensively examine the neural mechanism of cognitive impairment in patients with T2DM.

Many neuroimaging studies have used different methods to explore the patterns of abnormal brain structure and function in patients with T2DM under different cognitive states. Voxel-based morphometry (VBM) analysis has found reductions in cortical/subcortical gray matter and some overlapping brain regions in T2DM patients with and without MCI, whereas gray matter volume (GMV) atrophy is more widespread in T2DM patients with MCI (DMCI) (9, 10). Li et al. (10) suggested that subcortical atrophy may play a pivotal role in the cognitive impairment of patients with T2DM. Furthermore, several studies using different methods found slight changes in the white matter microstructure (11–13) and the white matter structure network (14, 15) in T2DM patients with normal cognitive function (DMCN), whereas patients with DMCI have extensive white matter abnormalities, suggesting that white matter microstructural and structure network change might give rise to cognitive impairment in patients with T2DM. Although the previous studies have revealed extensive changes in the brain structure of patients with T2DM under different cognitive states and reached relatively consistent conclusions, the results of studies on brain function in different cognitive states of patients with T2DM are inconsistent. For example, an fMRI study (16) indicated that the brain regions that have abnormal neuron activity are mostly the same in patients with T2DM under different cognitive states. Another study (17) found that patients with DMCI exhibited more extensively altered regional homogeneity and impaired network organization relative to patients with DMCN, suggesting that there may be a compensation mechanism in the development of cognitive impairment in T2DM. Moreover, an analysis of brain network connectivity showed more extensive impaired intra-network and inter-network connectivity in patients with DMCI (18). Zhang et al. (19) demonstrated, using independent component analysis (ICA), that patients with DMCI exhibited weakened functional connectivity (FC) and reduced GMV within the salience network (SN), whereas patients with DMCN exhibited enhanced intra-SN functional coupling, which suggests that changes in intra-SN FC and GMV were non-linear and complex in patients with T2DM and cognitive impairment. However, few studies have focused on changes in the patterns of brain hub nodes and their effects on the FC of the whole brain of patients with T2DM under different cognitive states. Previous studies have used degree centrality (DC) analysis to investigate the abnormalities of hub nodes in patients with T2DM, and their results suggested these abnormalities may reflect the aberrant communication of information in the brain of patients with T2DM (20, 21). Nonetheless, at the same baseline level, changes in patterns of hub nodes and their FC in the whole brain of patients with T2DM under different cognitive states are still unclear.

Degree centrality (DC) is a powerful measure that reflects abnormalities in the most connected hub nodes in functional networks (22, 23); this method can avoid the deviations caused by selecting brain regions based on prior hypotheses. In addition, as the brain is a complex system, it is essential not only to look at the individual regions but also to explore how brain networks work together through signal coherence (24). Brain network dysfunction and abnormal neuronal activity are the basis of cognitive impairment, and combing DC with secondary seed-based FC analysis can effectively detect abnormal neural network connections (25, 26).

In this study, we used both DC analysis and seed-based FC analysis to reveal the relationships among hub nodes and related network dysfunction and cognitive impairment in patients with T2DM. The key neuropathological features of cognitive impairment in patients with T2DM are advanced glycation end-products and amyloid-beta deposition, and some studies indicate that the cognitive dysfunction caused by amyloid-beta deposition is a non-linear process (27, 28). Based on previous fMRI studies and the neuropathological basis of cognitive impairment, we speculated that the patterns of brain functional changes in patients with T2DM under different cognitive states may be complex and diverse, but abnormalities of brain hub nodes and functional connections may be more extensive in patients with DMCI. Furthermore, these abnormally altered hub nodes and functional connections may correlate with clinical/cognitive variables.

## Materials and methods

### Participants

Sixty-seven patients with T2DM were recruited for this study from May 2019 to July 2020 at Shaanxi Provincial People's Hospital, including 33 patients with MCI (DMCI group) and 34 patients without MCI (DMCN group). Thirty-four healthy controls (HCs) were recruited from the health examination center of our hospital. All participants were between 45 and 70 years of age, right-handed, and had at least 6 years of education. The diagnostic criteria of T2DM were based on the American Diabetes Association's 2014 guidelines. Patients with T2DM were on stable therapy (diet, oral medications, and/or insulin). Patients with T2DM were excluded who had a history of hypoglycemia (blood glucose concentration of < 3.9 mmol/L) or hyperglycemia (blood glucose concentration of >33.3 mmol/L). The inclusion criteria of the DMCI group were: (1) complaints of hypomnesis, which occurred after a clinical diagnosis of T2DM; (2) an MMSE score >24 and MoCA score < 26; (3) no other physical, mental, or neurological disorders that could lead to cognitive impairment. The exclusion criteria for all the participants were: (1) severe claustrophobia or contraindications for MRI; (2) alcoholism, Parkinson's disease, major depression, brain injury, epilepsy, or other neurological or psychiatric disorders; (3) any other systemic disease.

Every participant arrived at the department for MRI at 6:30–7:00 p.m. after dinner and controlled their blood glucose according to their doctor's orders on the day of the scan. MRI was performed after ~30 min of structured clinical interview and a series of psychological tests. The test procedure and scan time of the HCs were the same as those of patients with T2DM. Only one participant was scanned each day to ensure that each participant completed the examination with relatively stable blood glucose. The study was approved by the ethics committee of Shaanxi Provincial People's Hospital, and written informed consent was obtained from all the participants. All methods were performed in accordance with relevant guidelines and regulations according to the Declaration of Helsinki.

### Clinical data and neuropsychological test information

All participants underwent the following neuropsychological examinations: Mini-Mental State Examination (MMSE), Montreal Cognitive Assessment (MoCA), Clock-Drawing Test (CDT), and Trail-Making Test A (TMT-A). TMT-A was used to test information processing speed and attention, and the increased time patients spend on the TMT-A test indicates that their attention and psychomotor speed are abnormal (29). CDT is often significantly associated with visuospatial and executive function, so it is commonly used to assess these cognitive domains (30). The neuropsychological tests were performed by a psychiatrist with more than 5 years of experience. HCs data were collected from the outpatient medical examination center. The participants' medical history and clinical data, including blood pressure, height, weight, and body mass index (BMI), were obtained from medical records and questionnaires. In addition, glycated hemoglobin (HbA1c), fasting blood glucose (FBG) concentration, triglyceride concentration, cholesterol concentration, and low-density lipoprotein cholesterol concentration were measured by standard laboratory tests.

### Image acquisition

The MRI images were acquired using a 3.0T MR scanner (Ingenia, Philips Healthcare, The Netherlands) with a 16-channel phased array head coil. Routine T2-weighted and fluid-attenuated inversion recovery (FLAIR) sequence (slices = 18, thickness = 6 mm, gap = 1 mm) were used to exclude visible brain lesions. The age-related white matter change scale (31) was used to evaluate lacunar infarcts and white matter hyperintensity based on FLAIR images; participant with a score >2 was excluded. Resting-state functional MRI (rs-fMRI) images were acquired using a gradient echo planar sequence with the following parameters: repetition time (TR) = 2,000 ms, echo time (TE) = 30 ms, flip angle (FA) = 90°, field of view (FOV) = 230 mm × 230 mm, matrix = 128 × 128, slices = 34, thickness = 4 mm (no gap), axial interleaved acquisition, and 200 volumes were acquired in each scan. Sagittal 3D T1-weighted images were acquired using a fast spoiled gradient echo sequence with the following parameters: TR = 7.5 ms, TE = 3.5 ms, FA = 8°, FOV = 250 mm × 250 mm, matrix = 256 × 256, slice thickness = 0.55 mm (no gap), and 328 sagittal slices. All participants were instructed to close their eyes and stay awake throughout the scan.

### Preprocessing of MRI data

The rs-fMRI data were preprocessed by the DPABI (http://rfmri.org/dpabi/) software package (32). The first 10 time points were discarded to ensure the stability of the magnetic field. The slice-timing and realignment of head motion correction were performed for the remaining 190 time points. Participants were excluded from this study whose head motion >1.5 mm and/or translation >1.5° rotation. Moreover, the scrubbing method was used to reduce the effect of high-head motion (33). We calculated the frame-wise displacement (FD) and set an FD threshold for bad volumes as 0.2 mm. The bad volumes were scrubbed which the FD values were greater than 0.2 mm, as well as one forward volume and two back volumes of the bad volumes. Then, each bad volume was modeled as a regressor in the model regression (34). Then, the images were normalized to the standard Montreal Neurological Institute (MNI) space with an echo planar imaging (EPI) template (resampling voxel size = 3 mm × 3 mm × 3 mm). In addition, we regressed out 24 head motion parameters, the linear trend signal, cerebrospinal fluid signals, and white matter signals. Finally, a temporal-band filter (0.01–0.08 Hz) was used to regress the effect of physiological noise.

### Degree centrality analysis

Differences in the brain functional hubs were obtained by comparing the DC maps of the three groups. First, a voxel-based correlation analysis of whole-brain function was performed on the preprocessed fMRI data, and Pearson's correlations were calculated for the time course from each voxel to every other voxel in the whole brain to create a correlation matrix. Second, the binarized matrix was obtained by thresholding each correlation at *r* > 0.25 (35, 36). Then, these voxel-wise DC values were converted into a *z*-score matrix to improve normality. Finally, the zDC maps were further smoothed (FWHM = 6 mm) for statistical analysis.

### Seed-based functional connectivity analysis

A seed-based approach was used for FC analyzes of the spatial smoothing (FWHM = 6 mm) data. The peak MNI coordinates of significantly altered DCs between the DMCI and DMCN groups were selected as the center of spherical regions of interest (ROIs) with a 6-mm radius. Then, the time courses were extracted for the ROIs, and correlations were calculated between each ROI and every other voxel within the brain to obtain FC maps. These FC maps were converted to *z*-score maps using Fisher's *z* transformation to improve the normality.

### Statistical analysis

The chi-square (χ^2^) test was used to analyze the sex-based differences among the three groups. One-way analysis of variance (ANOVA) was used to analyze other demographic and clinical and cognitive scores. A two-sample *t*-test was used to compare disease duration between the DMCI and DMCN groups. These analyzes were performed using SPSS version 24 (IBM Corporation, Armonk, NY, United States), and *P* < 0.05 was considered to be statistically significant.

The statistical analyzes of DC and seed-based FC were performed in DPABI software. Analysis of covariance (ANCOVA) was performed to investigate the regions with significant differences in DC and FC among the three groups, with age, gender, and years of education as covariates (*P* < 0.001, uncorrected, cluster >50). The ANCOVA results were used as a mask, and *post-hoc* analyzes within the areas were performed to investigate pairwise between-group differences (FDR corrected, *P* < 0.05).

Correlation analysis of average zDC and zFC values of the brain areas with significant differences was extracted from the DMCI and DMCN groups. Then, significant partial correlations (Bonferroni corrected, *P* < 0.05/13) were performed between the zDC values, zFC values, and clinical scores, with age, gender, and years of education as covariates.

## Results

### Clinical and neuropsychological data comparison

Two patients with DMCI were excluded from the analyzes for excessive motion; thus, 31 patients with DMCI, 34 patients with DMCN, and 34 HCs were included in the analyzes. There were no significant group differences in gender, age, education, blood pressure, BMI, triglycerides, total cholesterol, or low-density lipoprotein. Compared with HCs, the two T2DM groups showed higher FBG and HbA1c scores. Analysis of the neuropsychological data revealed the DMCI group had poorer MMSE, MoCA, and CDT scores and higher TMT-A scores compared to the DMCN group and HCs (see Table 1).

**Table 1:** Demographic, clinical, and neuropsychological characteristics of the participants.

**Variable**	**DMCI (*n* = 31)**	**DMCN (*n* = 34)**	**HC (*n* = 34)**	***F*/χ^2^ value**	***P*-value**
Gender (male/female)	18/13	22/12	21/13	0.30	0.86^#^
Age (years)	56.09 ± 6.11	53.97 ± 7.59	53.48 ± 4.82	1.54	0.21
Education (years)	13.90 ± 2.24	14.00 ± 2.53	14.20 ± 3.62	1.17	0.33
Duration (years)	9.25 ± 5.39	9.44 ± 5.34	–	–	0.89^&^
Systolic BP (mmHg)	127.96 ± 16.98	126.91 ± 22.97	122.91 ± 9.68	0.78	0.46
Diastolic BP (mmHg)	82.16 ± 15.84	80.73 ± 11.21	81.65 ± 6.44	0.14	0.88
BMI (kg/m^2^)	24.74 ± 2.89	25.09 ± 2.74	24.21 ± 2.27	0.91	0.40
FBG (mmol/L)	9.32 ± 3.67	8.34 ± 3.92	3.96 ± 2.45	13.62	< 0.001^a^^,^ ^b^
HbA1c (%)	8.01 ± 2.74	8.42 ± 1.75	5.53 ± 0.58	21.04	< 0.001^a^^,^ ^b^
TG (mmol/L)	1.78 ± 0.17	1.69 ± 0.26	1.42 ± 0.16	0.77	0.46
TC (mmol/L)	4.43 ± 1.66	4.33 ± 1.39	4.17 ± 1.94	0.16	0.82
LDL (mmol/L)	2.58 ± 0.96	2.44 ± 0.82	2.46 ± 1.13	0.14	0.86
MMSE	26.45 ± 0.72	29.03 ± 1.06	28.73 ± 1.26	60.34	< 0.001^a^^,^ ^c^
MoCA	22.97 ± 2.04	27.26 ± 1.08	27.01 ± 1.67	90.33	< 0.001^a^^,^ ^c^
CDT	17.66 ± 5.90	21.94 ± 7.82	22.24 ± 5.63	4.92	0.01^a^^,^ ^c^
TMT-A	92.67 ± 29.51	72.73 ± 29.73	65.35 ± 28.28	7.63	0.001^a^^,^ ^c^
FD	0.08 ± 0.01	0.07 ± 0.01	0.07 ± 0.01	0.16	0.82
DR (*n*)	4	3	–	0.28	0.60^#^^,^ ^&^
DPN (*n*)	10	8	–	0.62	0.43^#^^,^ ^&^
DN (*n*)	8	6	–	0.64	0.42^#^^,^ ^&^

Data are presented as mean ± standard deviation or number (%) unless otherwise indicated.

BMI, body mass index; FBG, fasting blood glucose; HbA1c, glycated hemoglobin; TG, triglyceride; TC, total cholesterol; LDL-C, low-density lipoprotein cholesterol; MMSE, mini-mental state examination; MoCA, montreal cognitive assessment; CDT, clock-drawing test; TMT-A, trail-making test A; FD, framewise displacement; DR, diabetic retinopathy; DPN, diabetic peripheral neuropathy; DN, diabetic nephropathy.

# *P* for the χ^2^ test.

& *P* for the two-sample t-test.

a *post-hoc* paired comparisons showing significant differences between patients with DMCI and HCs.

b *post-hoc* paired comparisons showing significant differences between patients with DMCN and HCs.

c *post-hoc* paired comparisons showing significant differences between patients with DMCI and DMCN.

### Degree centrality analysis

**Figure 1:**
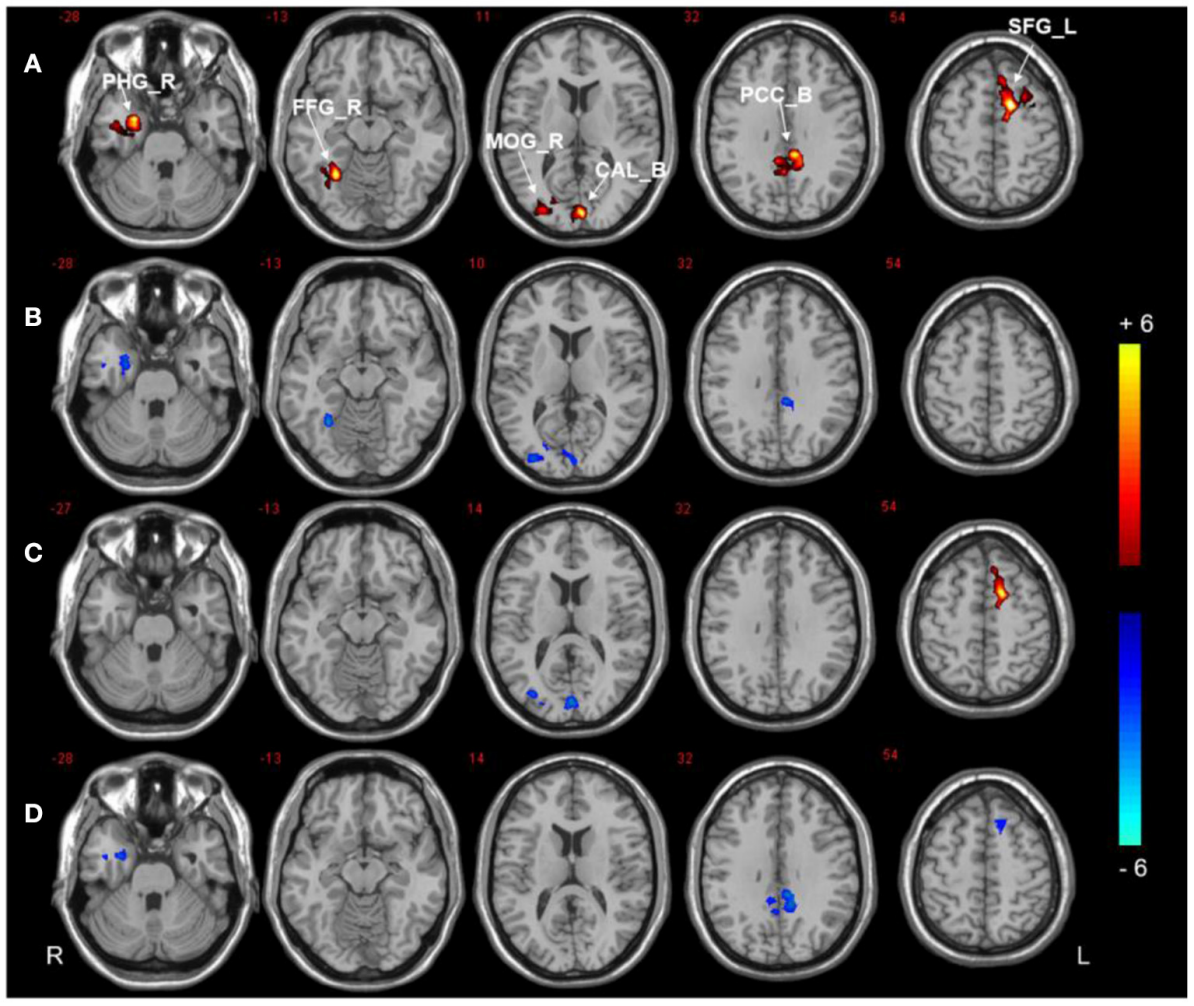
DC analysis of the three groups. **(A)** Significant difference in DC among three groups (*P* < 0.001, uncorrected, cluster >50). **(B)** Significant difference in DC between the DMCI and HC groups. **(C)** Significant difference in DC between the DMCN and HC groups. **(D)** Significant difference in DC between the DMCI and DMCN groups (*P* < 0.05, FDR corrected). Warm (cold) color indicates significantly increased (decreased) DC.

**Figure 2:**
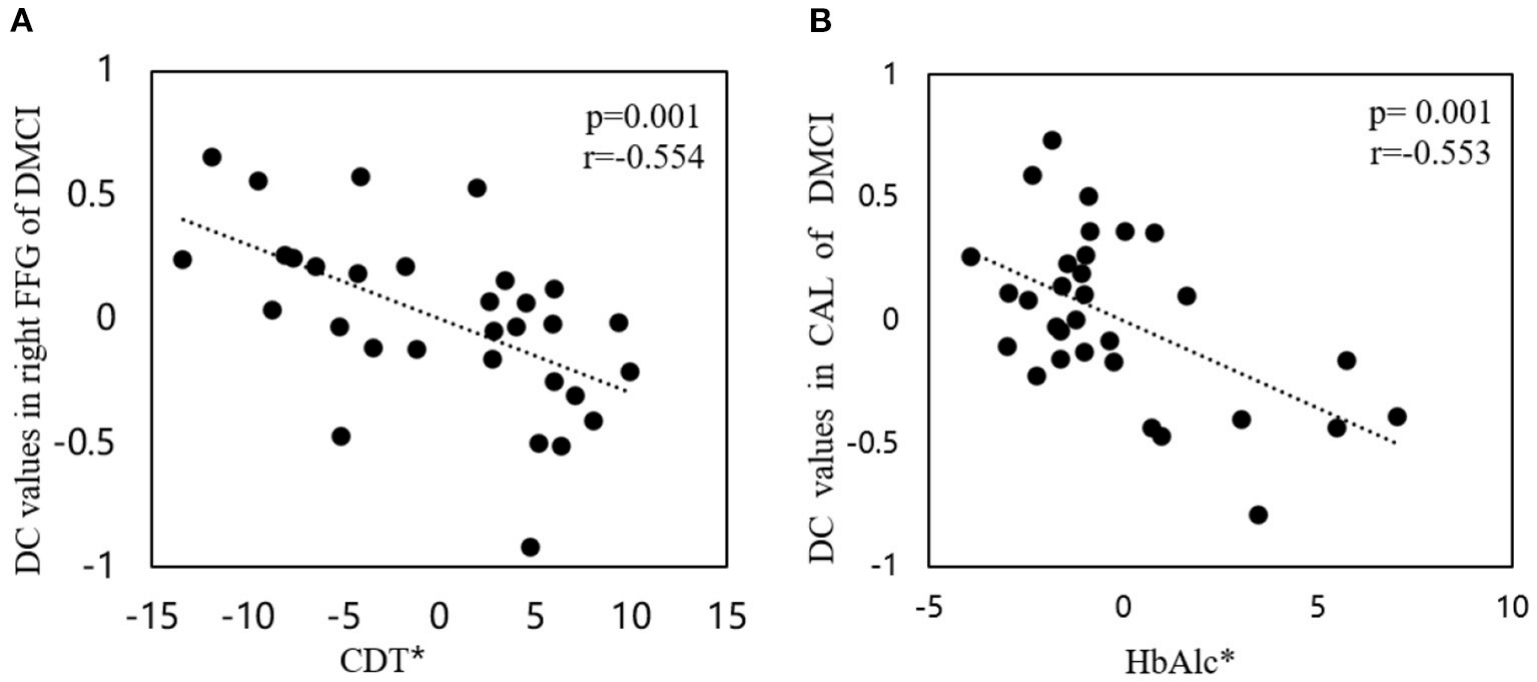
Correlations between DC values and clinical/cognitive variables. **(A)** Correlation between DC values in the right FFG and CDT scores in patients with DMCI (*r* = −0.554, *P* = 0.001). **(B)** Correlation between DC values in the bilateral CAL and HbA1c scores of patients with DMCI (*r*= −0.553, *P* = 0.001). The asterisk (*) indicates coordinate values, controlling for the effects of gender, age, and years of education.

Compared to the HCs, the DMCI group showed decreased zDC values in the right parahippocampal gyrus (PHG), fusiform gyrus (FFG), and middle occipital gyrus (MOG), the bilateral calcarine (CAL), and the posterior cingulate cortex (PCC), whereas the DMCN group showed increased zDC in the left superior frontal gyrus (SFG) and decreased zDC in the bilateral CAL and right MOG. Compared with the DMCN group, the DMCI group showed decreased zDC in the right PHG, bilateral PCC, and left SFG (see Table 2, Figure 1). In addition, the zDC values in the right FFG were negatively correlated with CDT scores of patients with DMCI (*r* = −0.554, *P* = 0.001) (Figure 2A). Moreover, the zDC values in the bilateral CAL were negatively correlated with the HbA1c scores of patients with DMCI (*r* = −0.553, *P* = 0.001) (Figure 2B).

**Table 2:** Significant differences in DC.

**Brain regions**	**Peak MNI**			**BA**	**Cluster size**	***F/T*-value**
	**X**	**Y**	**Z**			
**ANCOVA**
PHG_R	27	0	−30	36/28	69	15.27
FFG_R	30	−57	−15	19/37	64	15.30
CAL_B	−3	−90	9	17	102	16.48
MOG_R	30	−90	12	19/18	52	15.32
PCC_B	−6	−39	30	23	92	14.39
SFG_L	−15	3	54	8/6	183	18.51
**DMCI vs HC**
PHG_R	27	−6	−30	36/28	38	−4.86
FFG_R	30	−60	−12	19/37	44	−5.20
CAL_B	−3	−93	6	17	30	−4.59
MOG_R	30	−90	12	19/18	54	−5.29
PCC_B	−6	−39	30	23	27	−4.94
**DMCN vs HC**
CAL_B	−6	−93	11	18	47	−4.77
MOG_R	48	−75	3	19	30	−4.58
SFG_L	−12	16	54	8/6	116	5.05
**DMCI vs DMCN**
PHG_R	30	−6	−30	36/28	30	−4.51
PCC_B	−6	−39	30	23	68	−5.79
SFG_L	−6	15	54	8/6	24	−4.49

PHG, parahippocampal gyrus; FFG, fusiform gyrus; CAL, calcarine; MOG, middle occipital gyrus; PCC, posterior cingulate cortex; SFG, superior frontal gyrus; *F*-values refer to ANCOVA (*P* < 0.001, uncorrected, cluster >50) of DC analysis, *T*-values refer to *post-hoc* pairwise comparisons (FDR corrected, *P* < 0.05). L, left; R, right; B, bilateral.

### Seed-based functional connectivity analysis

**Figure 3:**
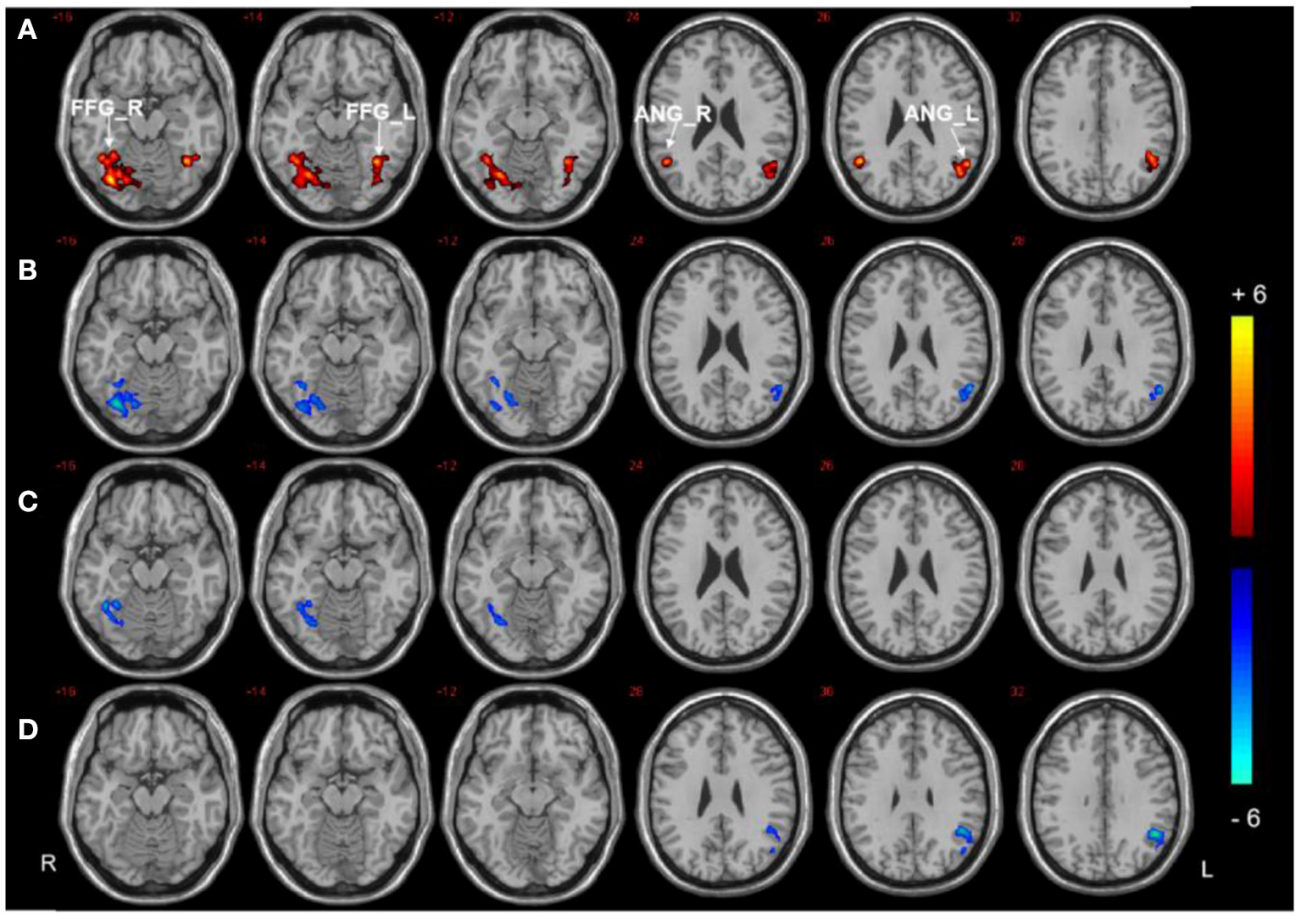
Seed-based FC analysis in the right PHG of the three groups. **(A)** Significant difference in FC among three groups (*P* < 0.001, uncorrected, cluster >50). **(B)** Significant difference in FC between the DMCI and HC groups. **(C)** Significant difference in FC between the DMCN and HC groups. **(D)** Significant difference in FC between the DMCI and DMCN groups (*P* < 0.05, FDR corrected). Warm (cold) color indicates significantly increased (decreased) FC.

**Figure 4:**
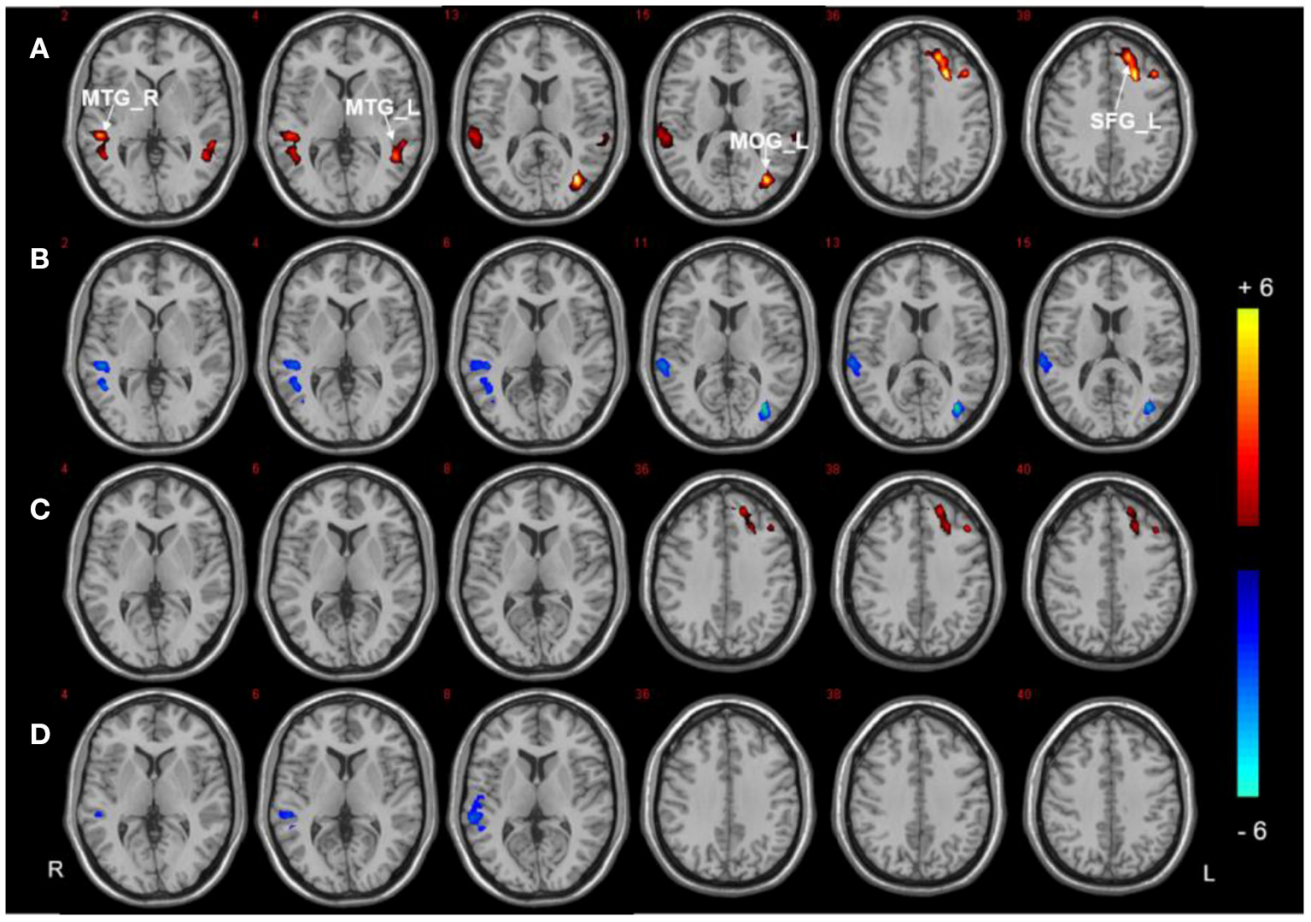
Seed-based FC analysis in the bilateral PCC of the three groups. **(A)** Significant difference in FC among three groups (*P* < 0.001, uncorrected, cluster >50). **(B)** Significant difference in FC between the DMCI and HC groups. **(C)** Significant difference in FC between the DMCN and HC groups. **(D)** Significant difference in FC between the DMCI and DMCN groups (*P* < 0.05, FDR corrected). Warm (cold) color indicates significantly increased (decreased) FC.

Three regions (the right PHG, bilateral PCC, and left SFG) were selected as ROIs to perform voxel-wise FC analyzes. The analyzes revealed between-group differences in the right PHG, as shown in Figure 3, Table 3. Compared to HCs, both T2DM groups showed decreased FC between the right PHG and FFG. The DMCI group also had significantly weaker FC values between the right PHG and left angular gyrus than the DMCN and HC groups. Moreover, FC analysis of the bilateral PCC showed reduced FC in the right middle temporal gyrus (MTG) and left MOG in the DMCI group, and the DMCN group showed increased FC in the left SFG compared with HCs. Compared to the DMCN group, the DMCI group showed reduced FC in the bilateral PCC and right MTG (Figure 4, Table 4). The seed-based FC analysis of the SFG found no significant differences among the three groups.

**Table 3:** Significant differences in FC in the right PHG.

**Brain regions**	**Peak MNI**			**BA**	**Cluster size**	***F/T-*value**
	**X**	**Y**	**Z**			
**ANCOVA**
FFG_R	34	−76	−17	37/19	207	17.61
FFG_L	−37	−58	−18	37	97	13.82
ANG_R	48	−57	24	39	63	13.20
ANG_L	−51	−61	32	39	94	14.66
**DMCI vs. HC**
FFG_R	36	−66	−18	19	106	−5.63
ANG_L	−54	−60	24	39	57	−4.95
**DMCN vs. HC**
FFG_R	33	−51	−18	19	49	−4.87
**DMCI vs. DMCN**
ANG_L	−48	−54	30	39	88	−5.19

FFG, fusiform gyrus; ANG, angular gyrus; SFG, superior frontal gyrus; *F-*values refer to ANCOVA (*P* < 0.001, uncorrected, cluster >50) of FC analysis in the right PHG, *T*-values refer to *post-hoc* pairwise comparisons (FDR corrected, *P* < 0.05). L, left; R, right.

**Table 4:** Significant differences in FC in the bilateral PCC.

**Brain regions**	**Peak MNI**			**BA**	**Cluster size**	***F/T*-value**
	**X**	**Y**	**Z**			
**ANCOVA**
MTG_L	−48	−54	3	22	53	11.94
MTG_R	61	−36	6	22	120	12.96
MOG_L	−33	−72	9	19	115	16.86
SFG_L	−24	24	36	9	104	14.72
**DMCI vs. HC**
MTG_R	51	−33	0	22	107	−5.50
MOG_L	−33	−72	9	19	87	−5.44
**DMCN vs. HC**
SFG_L	−17	43	36	9	65	5.05
**DMCI vs. DMCN**
MTG_R	60	−33	9	22	63	−4.50

MTG, middle temporal gyrus; MOG, middle occipital gyrus; SFG, superior frontal gyrus; *F*-values refer to ANCOVA (*P* < 0.001, uncorrected, cluster >50) of FC analysis in the bilateral PCC, *T*-values refer to *post-hoc* pairwise comparisons (FDR corrected, *P* < 0.05). L, left; R, right.

## Discussion

In our study, we combined DC analysis and voxel-wise FC analysis to explore abnormal hub nodes and the FC patterns of these hubs in patients with T2DM under different cognitive states. There were two main sets of results. First, decreased DC values were found in the visual cortex of both T2DM groups, and the DMCI group showed more extensive alterations of brain hub regions in the right PHG, bilateral PCC, and left SFG regions than in the DMCN group. Second, the seed-based FC analysis further suggested that there are functional connectivity disturbances in visuospatial and memory-related brain connectivity in patients with DMCI.

### DC alterations

We found decreased DC values in the bilateral CAL and right MOG in both T2DM groups. These two regions are thought to be involved in processing visual information and encoding visual memories (37). Visual function impairment exists in the early stage of patients with T2DM (38, 39) and long-term hyperglycemia is the main factor leading to visual cognitive impairment (40, 41). The correlation results of the present study also suggested that the functional abnormalities of bilateral CAL may be affected by HbA1c in patients with DMCI. In addition, we found that patients with DMCI exhibited decreased DC values in right FFG and correlated with worse CDT scores. Those evidence suggest that visuospatial impairment is more widespread in patients with DMCI. The right FFG is involved in visuospatial function (42), and alterations in the FFG can cause visual cognitive impairment in patients with MCI, especially facial cognition (43, 44).

The brain regions of PHG, PCC, and SFG are the core nodes in declarative memory (45), default mode network (46), and executive control functions (47), respectively. These brain regions play an important role in maintaining higher cognitive functions. Chronic hyperglycemia can affect hub nodes and disrupt the topological integration of the brain (48), which may be the reason for the decreased DC values of these nodes in the DMCI group compared with the DMCN group. The results of this study suggest that patients with DMCI have more abnormal higher cognitive function brain regions. Interestingly, compared with HC, the DC value of SFG in the DMCN group increased, which may suggest that patients with T2DM had abnormal changes in brain function before MCI. Multiple previous studies have found increased FC in the SFG in patients with subjective cognitive impairment (49, 50). The different patterns of changes in the SFG in the two groups of patients with T2DM seem unusual, but this may suggest that the changes in the SFG under different cognitive states may be a complex process. Multiple studies have demonstrated that the impairment of brain function may not be a linear process (51, 52), which may be the reason why the SFG was not significantly different among groups in subsequent FC analysis. In addition, the present study also found that patients with DMCI showed worse psychomotor speed and attention, which is consistent with previous findings (53, 54) that patients with MCI are often accompanied by the impairment of multiple cognitive domains.

### FC alterations in the right PHG

In our study, both T2DM groups showed reduced connectivity between the right PHG and right FFG. The FFG connects with the medial temporal lobe, including the PHG, which is related to visual working memory and visual-spatial abilities (55). A neuropathological study suggested that the Tau protein levels in the FFG and PHG were associated with longitudinal visuospatial cognitive decline in patients with AD (56). The pathological mechanism of cognitive impairment in T2DM is similar to that of AD (57), which may suggest that the disordered functional connectivity of PHG and right FFG is related to the abnormal visuospatial function of patients.

The angular gyrus is connected to both the PHG and hippocampus via the inferior occipitofrontal fasciculus (58, 59), and it plays a critical role in memory retrieval (60, 61). The angular gyrus, hippocampus/parahippocampal gyrus, and dorsomedial prefrontal cortex are activated during episodic autobiographical memory (62). Moreover, the parahippocampal region exhibits strong connectivity with the angular gyrus during episodic memory retrieval processing (63). Studies (2, 64) suggest that episodic memory dysfunction exists in patients with T2DM. The current study found that the DMCI group showed lower FC values in the right PHG and left angular gyrus than the DMCN and HCs groups, suggesting that patients with DMCI may have a dysfunction in episodic memory retrieval.

### FC alterations in the bilateral PCC

Research has confirmed that the spontaneous activity of the MTG is strongly correlated with semantic processing efficiency (65). Patients with MCI showed less effective connectivity between the MTG and PCC, and this impaired connectivity is correlated with cognitive performance during auditory verbal learning (66). Our study found the PCC showed reduced FC with the right MTG in the DMCI group. This is consistent with previous findings (67, 68), which may suggest abnormal semantic memory functioning in patients with DMCI.

The MOG is involved in visual processing, and patients with MCI show reduced connectivity between the PCC and MOG during visual cognitive tasks, which is associated with impaired visual processing (69). Consistent with the previous DC results, FC analysis further confirmed that visual impairment was more severe in patients with DMCI. In addition, similar to previous research (67), we found increased FC values between the PCC and left SFG in the DMCN group, which may be interpreted as a compensatory increase in patients' cognitive control of food. The SFG and PCC/precuneus play important roles in the cognitive control circuit, supporting cognitive control of appetite upon encountering visual food cues. It was found that neuropeptide oxytocin (OXT) was able to reduce food craving trends under cognitive control conditions and that the effects of OXT paralleled increased activity in the SFG, precuneus, and cingulate cortex (70).

### Limitations

This study has several limitations. First, this experiment is a cross-sectional study with a small sample size, and a larger sample could increase the credibility of our results. Second, although we adopted a relatively loose correction method in the ANCOVA analysis, the *post-hoc* analysis adopted strict FDR correction, which could minimize the false positive of between-group differences. Third, many of the patients in this study received various medications that may have biased the results, but that would be hard to avoid. Finally, relatively few behavioral scales were used in this study, which leads to the lack of behavioral support for some results. We will employ more abundant and comprehensive behavioral scales in future studies.

## Conclusion

This study found that patients with T2DM with different cognitive states have abnormal hub nodes and functional dysfunction. The brain area of hub nodes and functional dysfunction in patients with DMCI is more extensive and mainly located in vision and memory-related brain regions. Visual-related regions dysfunctions and disconnection may be involved in the neuropathology of visuospatial function impairment in patients with DMCI. This study provides valuable insights into the neurological underpinnings of T2DM-related cognitive impairment.

## Data availability statement

The original contributions presented in the study are included in the article, further inquiries can be directed to the corresponding authors.

## Ethics statement

The studies involving human participants were reviewed and approved by the Ethics Committee of Shaanxi Provincial People's Hospital. The patients/participants provided their written informed consent to participate in this study.

## Author contributions

YH and DZ drafted the manuscript and designed the experiment. YH performed the statistical analysis. XinZ, MC, and ZY collected the data. MT and JG contributed to performing the experiments and revising the manuscript. KA provided technical support. XL and XiaZ made contributions to the design of the experiment and revised the manuscript. All authors read and approved the final manuscript.
